# Collectanea Medica: Consisting of Anecdotes, Facts, Extracts, Illustrations, &c.

**Published:** 1822-07

**Authors:** 


### COLLECTANEA MEDICA:

CONSISTING OF

### ANECDOTES, FACTS, EXTRACTS, ILLUSTRATIONS, &c.

***Relating to the History or the Art of Medicine, and the Collateral Sciences.***

---

Floriferis, ut apes, in saltibus omnia libant,
Omnia nos, itidem, depascimur aurea dicta.

---

ART. I.—*Cases from a Paper entitled "Observations on the Disease usually termed Puerperal Fever:* by Dr. CAMPBELL, of Edinburgh." —(Edinburgh Medical Journal, April 1822.)

CASE I.—Mrs. N. æt. 25, a female of spare habit, but healthy constitution, fell in labour of her second child on Sunday 26th March, at five o'clock. At this time the uterine aperture fully equalled the size of a crown-piece, and felt quite soft and dilatable. The presentation was natural, the vertex presenting at the right acetabulum, and the face towards the left side of the pelvis; but uterine action was so trivial, that I deemed constant attendance unnecessary. On this account I took my leave, desiring that I might be called when the pains, by their frequency and power, should indicate that the patient required more attention. I was accordingly sent for at eleven P.M., when I found uterine action so powerful, and the first stage of labour so far advanced, that I considered it my duty to remain by the patient until she should be relieved; particularly as the os uteri was so dilatable, and the passages throughout so well prepared. Labour gradually advanced from this period, without any remarkable occurrence, until four in the morning, when the membranes gave way, and a limited quantity of liquor amnii was discharged. The head at this time had made very little progress through the brim, although the labour-pains had all along been strong. This little advance of the head was owing to its size, which, after the rupture of the membranes, was discovered to exceed the ordinary dimensions, while the pelvis did not seem to be more than usually capacious. The head, greatly elongated, was ultimately expelled at half-past twelve at noon, and the body and secundines soon followed. The fetus proved to be a male, considerably larger than the usual size. It was not convenient to have it weighed, but the large diameters of the head exceeded the usual dimensions by an inch and a quarter; and, to show that it was exposed to much pressure during its descent, the parietal protuberances were completely obliterated, being levelled with the rest of the skull. The patient had ℈j. of camphor in the evening, to allay after-pains and procure rest; and she continued to do well for the remainder of this and the following day, but continued talking incessantly, in defiance of every remonstrance.

*March 28th.*—I called at noon, and found the patient labouring under severe headach, deep-seated pain in the eyes, troublesome thirst, great heat of surface, and a strong full pulse of 135, with some diminution of uterine discharge. She informed me that she experienced severe rigors at two in the morning, which were succeeded by the uneasy feelings just detailed, and the diminution of the secretion of milk. The above symptoms were accompanied by excessive nausea, which sometimes ended in vomiting, the matter brought up having a bilious appearance. There was also pain above the pubis, but so little felt by the patient, that, if the question had not been asked her, it is probable she would not have mentioned it. When the hand, however, was applied over the abdomen, a little nearer the umbilicus than the pubis, the uterus could be felt much larger than it should be found at this period after parturition; and moderate pressure occasioned much pain. The tongue was clean and moist, but the bowels were torpid, attended with some pain in voiding the urine. The patient had a wild eye, and her countenance was equally unsettled. It was remarked that she made no inquiry after her child. Under circumstances like these, the practice was quite obvious; and I accordingly proposed immediate venesection, which was obstinately resisted by the patient, but under a promise to submit to it in the evening, unless relief were obtained by other remedies. In the mean time she had sub. mur. hyd. gr. iv. et ox. antim. c. phos. calc. gr. vi. made into a bolus with conserv. rosæ; and, in an hour afterwards, ℥j. of sulph. magnes. I likewise ordered warm fomentations to the abdomen, and occasionally small quantities of water-gruel, to promote the action of the bolus. In the evening, at six, I visited her, accompanied by Dr. Murphy: we then found the pulse at 140, and more contracted than at the forepart of the day. The cathartic medicines had produced several evacuations. The skin was somewhat moist; the lochia more copious; but there was no remission of the abdominal pain, which, instead of being confined to the uterus, as at the former visit, was now more diffused over the abdomen. She now submitted to be bled; and twenty-five ounces were taken from the arm, which induced syncope, and procured much relief.

*Thursday, 29th.*—Continued better in every respect; had slept well during the night; had very little uneasiness in the abdomen; lochia continued; but breasts were flaccid; pulse about 130. Diarrhœa followed the exhibition of the cathartic.—Hora somni tinct. hyoscyami ʒij. cum aq. menth. pp. ℥j. habeat.

*Friday, 30th,* 10 A.M.—Appeared dejected; pulse 145, and thready; tongue clean and moist, with its margins of a fiery red colour. When she attempted to sleep, her eyes remained half open; diarrhœa and the uterine discharge continued; abdominal pain was increased; the abdomen felt tumid, but not distended; and, when the umbilicus was pressed upon, the patient started up in bed from excruciating pain. At this time my friend Mr. Lizars saw the patient, and was of opinion that the lancet should again be used; which was done accordingly, and eighteen ounces of blood abstracted, with manifest relief. From the diarrhœa still continuing, she was now ordered some chalk julep. At one P.M. the abdominal pain recurred: twelve ounces more were detracted; the warm fomentations were continued; and a dose of the tinct. hyoscyam. exhibited at bed-time, with ℥ss. aq. acet. ammon. every half-hour while awake, with a view to promote diaphoresis.

*31st.*—I was called at 2 A.M. to attend another patient, whose labour was not concluded until six, prior to which time I had again been sent for to Mrs. N., from her becoming much worse; but the messenger, finding me from home, called Mr. Lizars, who, in consequence of the patient's aggravated sufferings, took away eighteen ounces more blood, with some alleviation of pain. Patient was very restless during the night, suffered greatly from nausea, and vomited twice something of a coffee-coloured substance; so that, from the very irritable condition of the stomach, she was unable to take the aq. ant. amen. oftener than twice. The tongue was still clean; the diarrhœa had been checked by the chalk julep, conjoined with aromatic confection; but the lochia continued. The abdomen was now nearly as large as it was previous to delivery, presenting a sugar-loaf appearance, and was not at all tense, but conveyed a feeling to the hand as if it contained air. At eleven A.M. I visited the patient, accompanied by Mr. Lizars and Dr. Murphy, who were of opinion that any further detraction of blood would be injudicious, although her sufferings were still excruciating. It was now resolved to apply sinapisms to the abdomen; and she supported the application of the sinapism for several hours, and thought herself relieved after it. In consequence of the abdominal pains having returned in a few hours afterwards, another sinapism was applied, but without any benefit. She was now ordered camphorated julep every second hour, with a view to sooth her sufferings, and support the vital powers. She vomited coffee-coloured matter frequently during the night, and sometimes talked incoherently.

*April 7th.*—She was seized at 3 A.M. with tremors, followed, at four, by cessation of pain, with coldness of the lower extremities, and clammy perspiration over the whole surface. She expressed an earnest desire to see me; and I was accordingly called at six, when I ordered small portions of brandy to be given occasionally. I saw her again at 8.30 A.M., at which time she was quite sensible; but the pulsation at the wrist had ceased, from which it was but too evident that death was near at hand. She accordingly sunk at eleven A.M.

*Dissection.*—On dividing the abdominal integuments, we were led to remark their extreme thinness, partly from having been stretched during gestation, and partly afterwards by the intestines in a state of distension. The recti muscles were at least three inches apart, remarkably thin, and greatly expanded. The epigastric artery was carried outwards on each side by the rectus muscle. On exposing the peritoneum, this membrane appeared unusually supplied with blood-vessels, and had contracted adhesions to the intestines. When the peritoneum was reflected, a white arborescent appearance was beautifully displayed on its intestinal surface, which, when further examined, was found to be the abdominal nerves greatly enlarged,—a circumstance never before noticed in such cases. When the abdominal viscera were brought into view, we had additional proofs of the propriety of our method of treatment; for the omentum, mesentery, and all the intestines, exhibited the most indubitable proofs of violent inflammation. The rectum, bladder, uterus, and vagina, were also affected with inflammation to a great extent. The body of the uterus appeared red in many points, as if a minute injection had been thrown into its vessels. The uterine ligaments and tubes were inflamed, but the ovaries appeared unaffected; and, in the left ovarium, the corpus luteum was quite conspicuous. There was no gangrene to be observed, this having been prevented by the freedom with which the lancet had been used. The escape of fetid gas from the abdominal cavity, as remarked by Hulme, was not observed in this case; and it appears to me that such an occurrence will not be met with except in cases attended with gangrene. The intestines, however, both great and small, were unusually distended with air; and to this the great size of the abdomen was to be attributed. The intestines were glued together by coagulable lymph, and there was effusion into the cavity of the abdomen, though inconsiderable,—not amounting in the whole to above eight ounces: it had more the appearance of purulent matter than serum.

Case IV.—A very nervous married female, aged 32, was delivered, by myself, of her fifth child, a male, at 5 A.M. on the 5th of April, after a very easy, natural labour. She continued well until three the following morning, when she was seized with severe rigors, and other concomitant symptoms. I did not see her until six in the evening; but she had been prescribed a dose of ol. ricini and warm fomentations by a female attendant. When I saw her, she had a strong full pulse of 145; she was lying upon her back, quite listless, and indifferent about her infant and other surrounding objects; she had severe nausea, but no vomiting; and complained much of difficult respiration. The abdomen, generally, was painful, particularly upon pressure; the uterine discharge was diminished, but not suppressed; and there was difficulty in voiding the urine. In this, as in all the other cases, there was pain over the forehead and in the eyes, and there was also much depression of spirits: in fact, the symptoms altogether predicted a disease of the most formidable nature. I immediately tied up the arm, and abstracted from a large orifice, in a full stream, thirty-two ounces of blood by weight, which induced a tendency to syncope, and diminished greatly the abdominal pain. I directed the warm fomentations to be continued, the following powder to be taken immediately, and to be repeated in the course of the evening, if necessary.—℞. Pulv. antim. gr. iij.; sub. mur. hyd. gr. ij. M. I saw the patient again at 9 P.M., and, from the state of the abdomen and condition of the pulse, I took away eighteen ounces more blood. The powder not having operated, she was ordered ℥j. sulph. magnes. and a cathartic enema at midnight, should the bowels still continue to resist the medicines previously administered.

*April 6th.*—In consequence of the state of the pulse and abdomen, twelve ounces more blood were detracted; and, at six in the evening, other eighteen ounces.

*7th.*—Patient felt much better in every respect.

*8th.*—Patient talked greatly during her sleep, vomited twice coffee-coloured matter, but, upon the whole, continued free from pain.

*9th.*—Continued nearly as the day before.—℞. Sulph. magnes. ad. ℥j. statim.

*10th.*—Could not void her urine this morning without the catheter; pulse 132, and full; lochia continuing; abdomen free from pain. Six P.M. pulse as in the morning; complained of acute pain in the abdomen, particularly about two inches above the pelvis, and in both iliac regions. V. S. B. statim ad ℥xxiv.; ordered to have twenty-four leeches applied to the pained parts.—9 P.M. V. S. B. ad ℥ix. Repet. An emollient cataplasm to be applied over the bites of the leeches, to promote bleeding.

From this time the patient began to recover, and she was quite well on the 18th. Mr. Lizars, Dr. Moore, Dr. Murphy, and Mr. Harland, all saw this patient. Ten days after her recovery from this complaint, she had a severe attack of phlegmasia dolens, of which she also recovered; and in six weeks after, notwithstanding all her sufferings, she was able to nurse her own child.

CASE V.—A stout, healthy, young married female, of 22 years of age, was delivered by myself of her second child, a male, after a very easy labour, on the 7th of April. On the 8th, she was visited by a female friend, to whom she mentioned that she was much troubled with after-pains; and she was advised to take four large wine-glassfuls of undiluted spirits to relieve them. In less than two hours after, she was seized with rigors of unusual severity, which were soon followed by symptoms not less severe, similar to those described in the other cases. I was called at ten P.M., and bled her to the amount of eighteen ounces, which induced complete syncope, and greatly relieved her uneasy feelings. I then ordered two small doses of sub. mur. hyd. and ox. antim. c. phos. calc. as in the former cases, and warm fomentations to be applied to the abdomen.

*9th.*—9.30 A.M. felt scarcely any pain; pulse 76; skin moist and temperate; lochia copious.

*10th.*—Continued as the day before. Ten P.M. was again called in a great hurry, in consequence of all her symptoms having returned with redoubled violence; which relapse was ascribed to her having been much agitated by her eldest child falling down stairs. I found her in great agony, unable to turn to either side in bed, with all the other symptoms equally as severe as in any of the former cases; the lochia continued, but the mammæ were flaccid. At this visit I took away about thirty ounces of blood, which produced complete syncope; after this she was ordered to take ten drops of the tinct. digit. purp. every hour and a half.

*April 11th.*—Six A.M. had still much pain of abdomen, with considerable tumefaction; took the digitalis five times in the course of the night, without producing any effect on the pulse, which this morning beat from 140 to 145. At this visit I abstracted eighteen ounces of blood, producing syncope in the recumbent posture; during which the patient vomited large quantities of coffee-coloured matter. At one P.M. the abdominal pain and velocity of the pulse continued, accompa-

1

nied with hurried and oppressed respiration: I therefore detracted twenty-four ounces more blood. At eight P.M. the pulse, if any thing, slower; tongue much cleaner; abdomen easier, and less tumid. Digitalis to be discontinued.

*April* 12*th.*—Ten A.M. continued nearly as yesterday; vomited much coffee-coloured matter during the night. Enema commune statim, et haust. efferves. ad ℥j. quaque secunda hora habeat. Two P.M., Ever since the exhibition of the enema, and two doses of the effervescent mixture, she vomited immense quantities of coffee-coloured matter, which was brought up almost without an effort. Eight P.M. Patient was scarcely able to speak; vomiting had ceased; pulse not very distinct at the wrist; the extremities were becoming cold, which, with the appearance of the countenance, indicated approaching dissolution. She continued sensible to the last, and expired at eleven P.M. apparently without much suffering.

*Dissection.*—When the body was exposed, we observed *vibices* on the lower extremities. On dividing the abdominal integuments, there did not appear to be much loss of substance, for they were by no means so thin as in Mrs. N.'s case; nor was the abdominal swelling so considerable. When the peritoneum was divided and reflected, it had not, as in Mrs. N.'s case, contracted any adhesion with the viscera underneath; and its vascularity, although somewhat preternatural, was not, however, very remarkable. Its nerves were greatly increased in size, as in Mrs. N.'s case. The vascularity of the mesentery was somewhat increased, but not to any great extent. The uterus and its appendages were the parts which suffered most; and here there were sufficient evidences of injury. The peritoneal coat of the uterus was greatly inflamed; and the broad and round ligaments still more so. The ovaries were enlarged to the size of a hen's egg, and the left ovary in a state of suppuration and ulceration; and both, when laid open, were found to contain sacs of purulent matter. There was effusion of serum into the cavity of the abdomen, but not to any extent; and it did not resemble pus so much as that found in the former case: it had more of a serous appearance, and contained curdy matter. The intestines did not cohere; nor were they interlined with coagulable lymph, as in the other case. The omentum, but particularly its inferior margin, was much inflamed, and its fat throughout greatly consumed. The stomach and intestines were much distended with flatus. Dr. Moir, Dr. Murphy, and Mr. Maxwell, witnessed this dissection.

